# Development and Characterization of a Pilot-Scale Model Cocoa Fermentation System Suitable for Studying the Impact of Fermentation on Putative Bioactive Compounds and Bioactivity of Cocoa

**DOI:** 10.3390/foods8030102

**Published:** 2019-03-19

**Authors:** Kathryn C. Racine, Andrew H. Lee, Brian D. Wiersema, Haibo Huang, Joshua D. Lambert, Amanda C. Stewart, Andrew P. Neilson

**Affiliations:** 1 Department of Food Science and Technology, Virginia Polytechnic Institute and State University, Blacksburg, VA 24060, USA; racinekc@vt.edu (K.C.R.); andhlee@vt.edu (A.H.L.); wiersema@vt.edu (B.D.W.); huang151@vt.edu (H.H.); 2 Department of Food Science, Pennsylvania State University, University Park, PA 16801, USA; jdl134@psu.edu

**Keywords:** *Theobroma cacao*, simulated pulp media, polyphenols, fermentation, procyanidin, catechin

## Abstract

Cocoa is a concentrated source of dietary flavanols—putative bioactive compounds associated with health benefits. It is known that fermentation and roasting reduce levels of native flavonoids in cocoa, and it is generally thought that this loss translates to reduced bioactivity. However, the mechanisms of these losses are poorly understood, and little data exist to support this paradigm that flavonoid loss results in reduced health benefits. To further facilitate large-scale studies of the impact of fermentation on cocoa flavanols, a controlled laboratory fermentation model system was increased in scale to a large (pilot) scale system. Raw cocoa beans (15 kg) were fermented in 16 L of a simulated pulp media in duplicate for 168 h. The temperature of the fermentation was increased from 25–55 °C at a rate of 5 °C/24 h. As expected, total polyphenols and flavanol levels decreased as fermentation progressed (a loss of 18.3% total polyphenols and 14.4% loss of total flavanols during fermentation) but some increases were observed in the final timepoints (120–168 h). Fermentation substrates, metabolites and putative cocoa bioactive compounds were monitored and found to follow typical trends for on-farm cocoa heap fermentations. For example, sucrose levels in pulp declined from >40 mg/mL to undetectable at 96 h. This model system provides a controlled environment for further investigation into the potential for optimizing fermentation parameters to enhance the flavanol composition and the potential health benefits of the resultant cocoa beans.

## 1. Introduction

Recently, cocoa (*Theobroma cacao)* and its putative bioactive compounds (particularly flavonoids) have been associated with various health benefits, including positive effects on cardiovascular, metabolic and endocrine diseases [1]. There is interest among health researchers, scientists, cocoa suppliers and manufacturers alike in tailoring the processing of cocoa to produce products with maximum health benefits. Three main groups of flavonoids exist within cocoas beans: proanthocyanidins (oligomeric and polymeric flavanols) constitute approximately 58% of the total phenolic content, followed by catechins (monomeric flavanols, ~37%) and anthocyanins (~4%) [2]. It is known that oxidation, condensation and other reactions that take place during cocoa fermentation and roasting reduce levels of native flavonoids, warranting investigation into how these reactions ultimately impact cocoa’s health benefits [3,4,5,6,7,8]. The widely-accepted assumption is that preservation of native flavonoids is critical for retaining bioactivity [9]. However, reactions (oxidation, epimerization, condensation, etc.) during processing may generate compounds with novel activities, potentially preserving or even enhancing health benefits [2,10,11,12,13] despite flavonoid loss. Recent findings by Ryan et al. [11] contradict the widely-accepted assumption that loss of native cocoa flavonoids corresponds with reduced activity in some cases. In their study, lower concentrations of flavonoids and total polyphenols in fermented cocoa products were not found to be associated with reduced bioactivity in in vitro digestive enzyme inhibition assays. These findings indicate the potential for optimization of processing factors such as fermentation and roasting to maximize the health benefits of cocoa.

Commercial cocoa fermentation is conducted on or in close proximity to the farm of origin in large heaps on the ground or in wooden boxes covered with banana leaves. Tremendous variability exists among on-farm heap fermentations, as differences in environmental microbiota, climate, substrate and fermentation methods all play key roles in microbial ecology and activity. These conditions are poorly documented and beans with differing fermentation histories are commingled into large batches, making post hoc evaluation of the impact of fermentation on bioactivity essentially impossible [8,12,14]. Furthermore, sourcing fermented beans for scientific research is difficult, and complex supply chains have left researchers with uncertainty on cultivar, processing parameters and heap consistency. Sourcing intact cocoa pods is also logistically challenging. With growing interest in the potential for optimization of processing to influence composition and subsequent bioactivity of cocoa, a controlled model pilot-scale fermentation system is needed. Employing dried, unfermented beans and simulated pulp media eliminates the needs for fresh unopened pods, a major limitation for cocoa fermentation research in regions distant from cultivation. Such a system would provide the ability to control all aspects of fermentation using the exact same starting beans, eliminating confounding variables when sourcing is not under full control of the investigators.

Some preliminary work has been done to explore controlling cocoa fermentation for research purposes (both for characterizing fermentation processes as well as modifying outcomes). Several laboratory bench-scale model fermentations have been conducted in a variety of vessels, including plastic [4,15] and stainless-steel [16], to examine the microbial influence and overall impact of starter cultures on cocoa fermentation. These model fermentations generally use pulp and beans from freshly harvested pods—which is not practical for frequent large-scale use in cocoa fermentation research conducted in non-tropical regions—and use inoculated and ambient pulp mediums [6,17,18,19,20,21]. Our group recently developed a large laboratory bench-scale fermentation using simulated pulp media and dried unfermented cocoa beans as starting material [22]. To our knowledge, ours was the first model of this scale. Along with other laboratory bench-scale model fermentation systems, this model can be used to evaluate the impact of controlled fermentation on putative bioactive compounds in cocoa. Fermentation can cause anywhere from 0–70% loss of total polyphenols, and following fermentation the beans are dried and roasted, causing an additional 15–40% loss [2,3,23,24,25,26,27,28,29,30,31]. Nonetheless, recent findings have indicated that lower concentrations of specific cocoa flavonoids and total polyphenols in a given product are not always associated with decreased bioactivity [11].

Additional studies employing a combination of analytical, in vitro and in vivo approaches are needed to advance the understanding of how specific cocoa flavonoid losses during cocoa processing affect the bioactivities of cocoa. Studies are then needed to optimize processing, including fermentation, to maximize the desirable health benefits of cocoa. However, to employ techniques that go beyond analytical characterization of the fermented product, such as in vivo bioactivity studies, larger amounts of experimentally fermented cocoa would be required than can be reasonably produced using existing laboratory bench-scale model systems (pilot scale: tens of kgs of fermented beans or more, instead of bench scale: hundreds of grams). Hence, our objective was to develop and characterize a pilot-scale model cocoa fermentation system suitable for studying the impact of fermentation on putative bioactive compounds. Dried, unfermented beans and a simulated pulp media and ambient microorganisms were used due to the limitations associated with sourcing and transporting fresh whole cocoa pods. Specifically, our hypothesis was that broad microbial and chemical changes similar to those generally observed in heap fermentations could be replicated in non-tropical regions by using a pilot-scale model system designed to simulate the conditions occurring in the middle of a well-turned cocoa heap, and that this system could be used to generate sufficient amounts of fermented cocoa beans for further research on the effect processing has on the potential health benefits of cocoa.

## 2. Materials and Methods

### 2.1. Chemicals and Standards

Citric acid, yeast extract, malt extract, calcium lactate pentahydride, tween 80, sodium hydroxide, magnesium sulfate heptahydride, manganese sulfate monohydride, sucrose, glucose, fructose, peptone, calcium carbonate and agar were obtained from Thermo Fisher Scientific (Waltham, MA, USA). Cycloheximide was obtained from MP Biomedicals, LLC (Solon, OH, USA) and oxytetracycline dihydrate was obtained from Acros Organics (Springfield Township, NJ, USA). Lactic acid, Folin–Ciocalteu reagent, 4-dimethylaminocinnamaldehyde (DMAC), (±)-catechin, (–)-epicatechin and procyanidin B2 (PCB2) were obtained from Sigma-Aldrich (St. Louis, MO, USA). Procyanidin C1 (PCC1) and cinnamtannin A2 (CinA2) were obtained from Planta Analytica (New Milford, CT, USA). Solvents were ACS grade or higher.

### 2.2. Pilot-Scale Fermentation Model

Raw unfermented Criollo cocoa beans (18 kg) (Natural Zing LLC, Mount Airy, MD, USA), sourced from Ecuador, were rehydrated in 66.4 × 44.3 × 34.3 cm plastic fermentation boxes (Polypropylene, Sterilite, Townsend, MA, USA) by submersion in approximately 20 L of distilled, deionized (DI) water for 24 h. The final moisture content of the beans after rehydration was 39.7% (IR-120 Moisture Analyzer, Denver Instrument, Bohemia, NY, USA), close to the lower end of the typical moisture range (40–60%) for fresh beans [32,33]. Rehydrated beans were then drained and 15 kg were mixed with 16 L of simulated pulp media, prepared as described by Lee et al. [22] with minor modifications. Simulated pulp media were obtained by mixing 3 separate solutions (solutions A, B and C). Solution A consisted of citric acid (10 g/L), yeast extract (5 g/L), peptone (5 g/L), calcium lactate pentahydride (1 g/L) and tween 80 (1 mL/L); the pH was adjusted to 3.6 using 1 N NaOH and then volume corrected to a total of 9.6 L before autoclaving (121 °C, 15 min) to ensure sterility of the medium. Solution B was a 4.8-L sugar solution, with 1.6 L each of sucrose (83.3 g/L), glucose (133.3 g/L) and fructose (150 g/L). Each sugar solution was autoclaved and cooled prior to fermentation. Solution C was prepared the day of fermentation and consisted of magnesium sulfate heptahydride (1 g/L) and manganese sulfate monohydride (0.4 g/L) for a total of 1.6 L. Boxes were loosely covered with their plastic lids and placed inside a pre-heated (25 °C) incubator (Forma 29 cu ft Reach-In Incubator, Model No. 3950, Thermo Fisher Scientific, Waltham, MA, USA). Scale-up from the bench scale was required to produce sufficient material for experiments evaluating the impact of fermentation on cocoa bioactivity using in vivo models. Two factors determined the scale of the pilot system: (1) the amount of fermented product required for animal studies using the fermented material as substrate, and (2) the size of the largest incubator available in our pilot plant. Taking these two factors into account, the batch size was increased by over 10-fold from a 1.2-kg batch of rehydrated beans at the bench scale to a 15-kg batch at the pilot scale. The bench-scale system was under constant agitation in a shaker/incubator, which was not feasible in this pilot system. To maintain maximum dissolved oxygen (DO) possible in a static system, fermentation vessel size and shape were selected to maximize the surface area to volume ratio, and a stirring regime was implemented wherein the contents of the vessel were well-mixed twice daily. Through preliminary work, this mixing regime, in combination with mixing due to gas evolution during fermentation, proved sufficient to maintain a well-mixed condition in the fermentation vessel and to support the microbial succession required for fermentation.

The fermentation was performed in duplicate, simultaneously under identical conditions (2 replicate boxes each employing the conditions described above: 15 kg rehydrated beans and 16 L simulated pulp per box) using ambient microorganisms (i.e., no inoculation) and took place over a period of 168 h (representing the upper end of the spectrum of reported heap fermentation times that occur on-farm). In the future, fermentation time can be varied to obtain different extents of fermentation. The incubator set point was raised 5 °C per 24 h to a final temperature of 55 °C in order to mimic temperature progressions seen in heap fermentation [3,8,34,35]. Beans were manually agitated for 3 min every 12 h to ensure that the simulated pulp media were aerated [14,34,35,36]. The agitation step was critical to ensure the expected succession of the microbial communities due to our model’s inability to introduce oxygen by draining pulp away, as typically happens during heap fermentations when the pulp is liquefied and the heap is manually turned. Pulp and bean samples were collected every 24 h throughout the fermentation. Pulp dissolved oxygen (DO) and pH values were monitored using benchtop meters (Orion DO Probe 083005MD; Orion Versa Star Pro pH meter; Thermo). After 168 h of fermentation, the beans were drained to remove the remaining pulp media, rinsed with water and spread evenly onto baking sheets. Beans were oven dried (Rational, Landsberg am Lech, Germany; Blodgett, Burlington, VT, USA) at 45–65 °C until the moisture content fell below 8%. These conditions mimicked typical commercial drying protocols [3,34,36,37]. After drying, beans were thoroughly mixed together and stored at 4 °C.

### 2.3. Microbial Enumeration

Enumeration methods and selective media were based on the protocols of Nielsen et al. [34] and Ho et al. [38] with minor modifications. Collected pulp media (5 mL) from each time point was diluted with 45 mL sterile 0.1% peptone water, and 1 mL of the resulting mixture was then diluted 10-fold. Aliquots (0.1 mL) were spread inoculated to nutrient agar appropriate for the growth of yeast, lactic acid bacteria (LAB) and acetic acid bacteria (AAB). Yeast cultures were spread on YM media (3 g/L yeast extract, 3 g/L malt extract, 3 g/L peptone, 10 g/L glucose, 20 g/L agar) with 100 mg/L oxytetracycline and incubated at 37 °C. LAB were cultured anaerobically by a BD GasPAK EZ gas generating system (Franklin Lakes, NJ, USA) on de Man–Rogosa–Sharpe (MRS) agar (Sigma) with 400 mg/L cycloheximide at 37 °C, and AAB were cultured on GYC media (50 g/L glucose, 10 g/L yeast extract, 30 g/L calcium carbonate, 20 g/L agar, pH = 5.6) with 400 mg/L cycloheximide by incubating at 25 °C. Bacterial enumeration was performed in duplicate on analytical replicates for each fermentation box and time-point. Colonies were counted and presented as log colony-forming units (CFU)/mL.

### 2.4. Cut Test

The cut test is the standard assessment of post-fermentation bean quality and suitability to move forward in processing [3,39]. A reduced sample-size version of the cut test was performed as follows: 6 beans from each 24-h sampling were cut through lengthwise and each half examined for color and quality defects. Although this test is not very applicable to low-anthocyanin beans like Criollo, a purple interior is indicative that the fermentation ended prematurely, while a brown interior is indicative of a successful fermentation [3,40].

### 2.5. Fermentation Index

Fermentation index (FI) monitors the color change within the bean cotyledon during fermentation. This color change is due to the decreasing anthocyanin content as beans progress through fermentation. FI was measured based on the method of Romero-Cortes et al. [41] with minor modifications. Five to seven randomly selected cocoa beans from each time point were frozen with liquid nitrogen and ground to a fine powder. A 50-mg sample of the resulting powder was weighed and mixed with 5 mL MeOH:HCl (97:3 *v*/*v*). Samples were extracted at 4 °C for 16–18 h on a rotating shaker, centrifuged for 5 min at 3500× *g*, and the supernatant was collected. Absorbance was measured using a BioTek Synergy 2 plate reader (BioTek, Winooski, VT, USA) on a 96-well plate (Corning Inc., Corning, NY, USA) at 460 and 530 nm. These wavelengths were chosen to express structural properties and distributions through fermentation, as 530 nm is a general λ_max_ for anthocyanin spectra and 460 nm reflects the glycoside distribution [42]. FI was determined based on the ratio of the absorbance at 460 nm compared to that of 520 nm.

### 2.6. Bean pH

Approximately 5–7 cocoa beans collected at each time point were frozen with liquid nitrogen, the shells were removed and the nibs were ground. Ground nibs (5 g) were collected and mixed with 100 mL hot water (90 °C) and stirred for 30 s. The cocoa water solution (25 mL) was then filtered through Whatman #4 filter paper and collected for pH analysis. It is important to note that this procedure was not for quantifying the actual pH of the cocoa bean itself, but rather to measure the acidity derived when bean acids are diluted into water; it is useful for comparison between the pH of solutions produced by beans at different time points.

### 2.7. High-Performance Liquid Chromatography (HPLC) Analysis of Fermentation Metabolites

#### 2.7.1. Pulp Media Sample Preparation

Pulp media samples were diluted 10-fold with distilled water, vortexed and centrifuged for 5 min at 5000× *g*. Next, 1 mL of supernatant was removed and filtered through a 0.45-µM polyvinylidene difluoride (PVDF) membrane filter (Thermo Fisher Scientific, Waltham, MA, USA) into vials.

#### 2.7.2. Bean Sample Preparation

Approximately 5–7 cocoa beans were peeled so that the nibs were exposed. Next, 5 g of nib were weighed and added to distilled water at a 10× dilution. The bean water solution was homogenized at high speed for 2 min in a blender (Waring Products, Calhoun, GA, USA). The blended mixture was then centrifuged for 5 min at 5000× *g* and 1 mL supernatant was removed and filtered into vials.

#### 2.7.3. Analysis

Bean and pulp samples were analyzed by HPLC on an Agilent HPLC 1260 Infinity Series (Agilent Technologies, Santa Clara, CA, USA) using an Aminex HPX-87H column (300 × 7.8 mm, 50 °C) (Bio-Rad Laboratories, Hercules, CA, USA) and a refractive index (RI) detector (35 °C). A 0.005 M H_2_SO_4_ isocratic mobile phase at a flow rate of 0.6 mL/min was used for analyte separation. The sample injection volume was 5 µL. Triplicate analytical replicates were prepared and analyzed from each fermentation time point. A standard curve was prepared with a range from 0.5 to 5.0 g/L. Sugars (sucrose, glucose, fructose), ethanol, glycerol and organic acids (acetic acid, lactic acid, succinic acid, citric acid) were quantified.

### 2.8. Polyphenol Extraction and Quantification

Whole cocoa beans (40 g) were frozen with liquid nitrogen and ground into a fine powder. To defat, the powder was mixed with 150 mL hexane and sonicated for 10 min at 22 °C. The mixture was then centrifuged for 5 min at 5000× *g*, the supernatant was discarded and then the process was repeated. Once defatted, the powder was allowed to dry at room temperature. Once dry, the powder was mixed with 150 mL extraction solution (70:28:2 acetone, water, acetic acid *v*/*v*/*v*), sonicated for 10 min at 22 °C and centrifuged for 5 min at 5000× *g*. The supernatant was collected, and this procedure was repeated three times for a total volume of 450 mL. All collected supernatant was pooled and placed under vacuum on a rotary evaporator at 40 °C until all acetone had evaporated. The resulting extract was freeze dried for 72 h and the yield was calculated. Total phenolic content (all polyphenols, including flavanols as well as other flavonoids and non-flavonoid phenolics) of both the nib and shell was determined by the Folin–Ciocalteu colorimetric assay and total flavanols (only catechins and proanthocyanidins) measured by the 4-dimethylaminocinnamaldehyde (DMAC) colorimetric assay, as previously described by Dorenkott et al. [10]. These values were expressed in mg gallic-acid equivalents (mg GAE)/g bean and mg PCB2/g bean, respectively.

### 2.9. Individual Polyphenol Analysis by Reversed Phase UPLC-MS

Monomeric catechins and low molecular weight procyanidins were measured by UPLC-MS on an Acquity H-Class UPLC-QDa Mass Detector (Waters Corporation, Milford, MA, USA). Cocoa extract (CE) was diluted with 0.1% formic acid (*v*/*v*) in water and 0.1% formic acid in acetonitrile (95:5 *v/v*), to a final concentration of 0.1 mg/mL, filtered (13 mm diameter syringe filters, 0.22 µm nylon membrane with propylene housing, Microsolv, Leland, NC, USA) into vials, and held at 10 °C. Samples were analyzed on an Acquity HSS T3 column (2.1 × 100 mm, 1.8 µm particle size) in combination with an Acquity HSS T3 VanGuard pre-column (2.1 × 5 mm column, 1.7 µm particle size) at 43 °C. Binary gradient elution was performed using 0.1% formic acid (*v*/*v*) in water (Phase A) and 0.1% formic acid (*v*/*v*) in acetonitrile (Phase B). The solvent flow rate was 0.6 mL/min and the linear gradient elution was as follows: 95% A (0–0.5 min), 65% A (6.5 min), 20% A (7.5–8.6 min) and 95% A (8.7–10.5 min). Samples were held at 4 °C and the injection volume was 10 µL. (–)-electrospray ionization (ESI) together with mass spectrometry (MS) was used to analyze the UPLC eluent. The ionization settings were as follows: (–) mode, 0.8 kV capillary voltage, 15 V cone voltage and 600 °C probe temperature. Triplicate analytical replicates were prepared and analyzed from each fermentation time point. Authentic standards of (±)-catechin, (–)-epicatechin, PCB2, PCC1 and CinA2 were utilized, and settings for selected ion response (SIR) monitoring of each compound are listed in Table 1. Data were collected using Empower 3 software (Milford, MA, USA).

### 2.10. HILIC UPLC-MS/MS

Monomeric flavanols and procyanidins were analyzed by hydrophilic interaction liquid chromatography (HILIC) UPLC-MS/MS as previously described [43]. A Waters Acquity H-class UPLC equipped with an Acquity Torus DIOL column (2.1 × 100 mm, 1.7 µL, 45 °C) and Torus DIOL VanGuard Pre-column (2.1 × 5 mm, 1.7 µL) were used for analysis. Gradient elution was performed with 2% acetic acid in acetonitrile (phase A) and 3% water and 2% acetic acid in methanol (phase B). Solvent flow rate was 0.8 mL/min and elution was carried out as followed: 100% A (0 min), 55% A (5.7 min), 5% A (6.0 min) and 100% A (6.7–9.0 min). The UPLC eluent was analyzed by (−)- mode ESI coupled to tandem mass spectrometry (MS/MS) on a Waters Acquity triple quadrupole (TQD). Aqueous ammonium formate (0.04 M, 5 µL/min) was added to the eluent flow stream post-column to enhance ionization. Ionization settings were as follows: (−) mode, capillary and cone voltages: −4.5 kV and 60.0 V, extractor voltage: 1.0 V, source and desolvation temperatures: 150 °C and 500 °C. N_2_ was used for the cone and desolvation gas at 50 and 1000 L/h, respectively. For MS/MS, Ar was the collision gas at 0.1 mL/min. Parent and signature daughter ions were subjected to multi-reaction monitoring (MRM) with a mass span of 0.2 Da and 1.0 sec of inter-channel delays and inter-scan times. Calibration curves for standards DP 1-9 (Planta Analytica, New Milford, CT, USA) were prepared and analyzed with dilutions ranging from 6.93 × 10^−7^ – 0.091 mg/mL. MRM settings for each compound are listed in Table 2. MassLynx software (version 4.1, Waters) was used to acquire data.

### 2.11. Data Analysis and Statistics

The fermentation was performed in duplicate, simultaneously under identical conditions. Analyses were performed on samples from each replicate; analytical replicates were averaged together to create a composite value for each fermentation replicate. Data from distinct time points were analyzed by one-way ANOVA to determine overall significance. If significant differences were detected, Tukey’s HSD post-hoc test was then used to compare all time point means. Significance was defined as *p* < 0.05. Analyses were performed using GraphPad Prism 7.03 (GraphPad, La Jolla, CA, USA).

## 3. Results

### 3.1. pH, DO, FI, Cut Test

**Figure 1:**
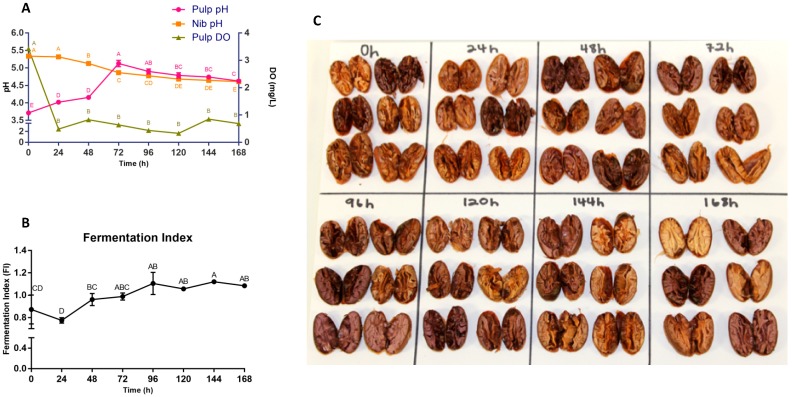
(**A**) pH measurements for both simulated pulp media and bean nib. It is important to note that, for bean nib measurements, these values do not quantify the pH of the cocoa bean itself, but rather of the acidity derived when bean acids are diluted in water. These nib values are useful for comparison between the pH of the solution produced by beans at different time points. Dissolved oxygen (DO) measurements within the simulated pulp media are expressed in mg/L. (**B**) Fermentation index (FI) expressed as a ratio of absorbance at 460 and 530 nm. (**C**) Cut test of six randomly selected beans per each timepoint. Beans were selected from both fermentation treatments to form one composite representation. Values are presented as the mean ± SEM of fermentation replicates. Significance between time points for each value was determined by one-way ANOVA and Tukey’s HSD post-hoc test (*p* < 0.05). Time points with different letters are significantly different within values.

As shown in Figure 1A, the mean pH of the simulated pulp media started at approximately 3.6, increased to a maximum value at 72 h and slightly declined to a final mean of 4.6. The solution made from the nib had a mean pH of 5.4, and the pH declined throughout fermentation to a final mean of 4.6. Initial DO levels were high due to the fresh mixing of simulated pulp and beans but decreased rapidly over the first 24 h followed by consistent levels for the remainder of the fermentation. It has been determined that cocoa mass is adequately fermented when FI measurements are ≥1 [41] and, as shown in Figure 1B, FI values began at 0.872 ± 0.065 and values ≥1 were initially achieved between 48–72 h. Figure 1C shows the cut test of beans selected at each time point. There were a variety of colors over the span of the fermentation, from light/dark brown to purple.

### 3.2. Microbial Enumeration and Fermentation Products

**Figure 2:**
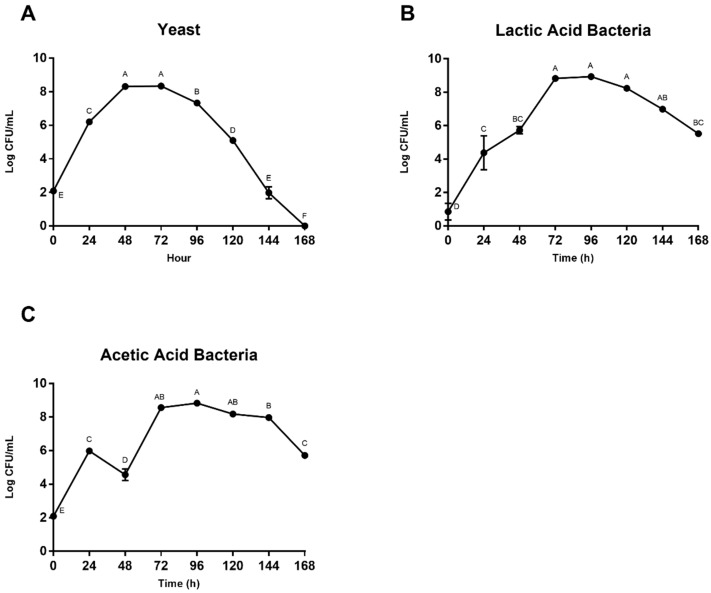
Enumeration of (**A**) yeast, (**B**) lactic acid bacteria (LAB) and (**C**) acetic acid bacteria (AAB) in simulated pulp media, expressed in log colony-forming units (CFU)/mL. Values are presented as the mean ± SEM of fermentation replicates. Significance between time points was determined by one-way ANOVA and Tukey’s HSD post-hoc test (*p* < 0.05). Time points with different letters are significantly different within values.

Microbial population changes are shown in Figure 2. Yeasts proliferated early (Figure 2A) with a 6-log increase over the first 48 h followed by a decline over the remainder of fermentation, ending with no measurable colonies. LAB (Figure 2B) presented a similar but less dramatic trend with approximately a 7-log increase over the first 72 h, followed by a moderate decline. AAB (Figure 2C) levels fluctuated before peaking at 72–96 h and then exhibiting a similar decline as LAB.

**Figure 3:**
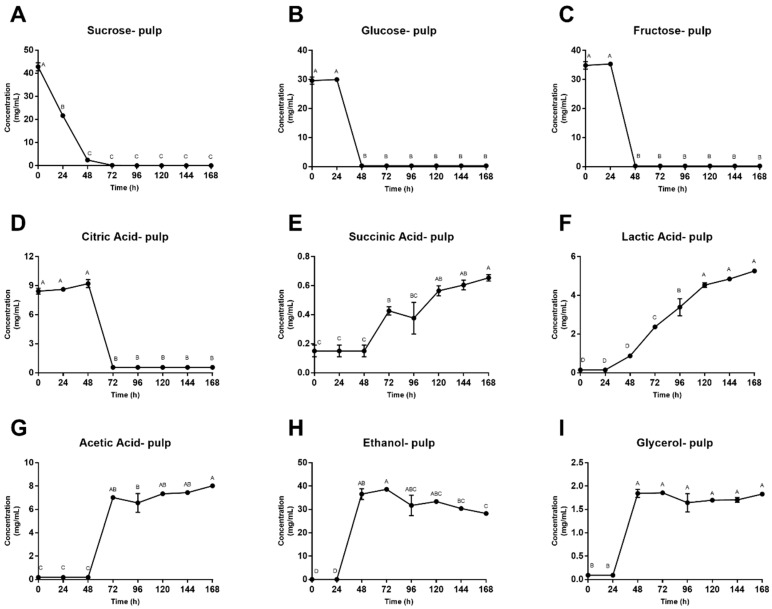
(**A**–**I**) Concentration of fermentation substrates and metabolites in simulated pulp media across 168 h. Values are presented as the mean ± SEM of fermentation replicates. Significance between time points was determined by one-way ANOVA and Tukey’s HSD post-hoc test (*p* < 0.05). Time points with different letters are significantly different within values.

Concentration of fermentation substrates and metabolites in simulated pulp media are shown in Figure 3. During the first 48–72 h of fermentation, sugar and citric acid concentrations dropped significantly and remained close to zero for the remainder of the fermentation. Contrarily, ethanol, glycerol and acetic acid remained relatively constant for the first 24–48 h of fermentation before demonstrating a dramatic increase in simulated pulp media concentrations. Succinic acid concentrations remained constant for the first 48 h followed by a 3-fold increase in simulated pulp media. Lastly, lactic acid was the only metabolite that showed a consistent increase throughout the entire fermentation, increasing almost 6-fold by the end of the 168 h.

**Figure 4:**
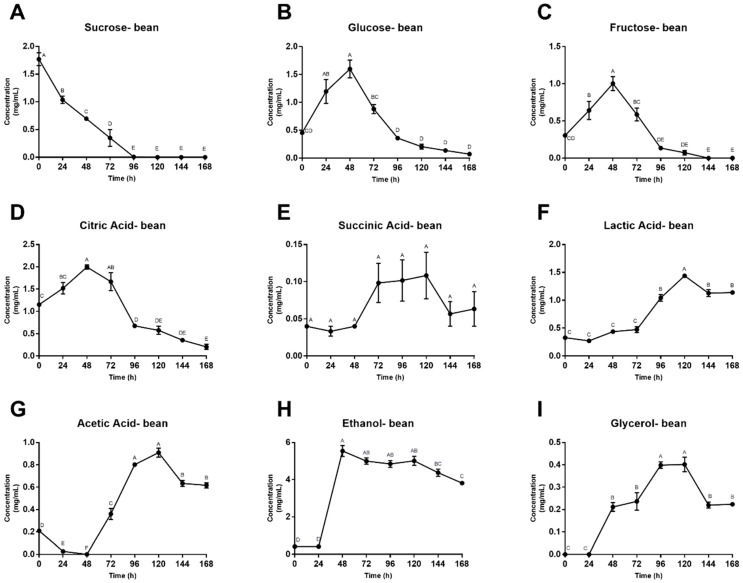
(**A**–**I**) Concentration of fermentation substrates and metabolites in cocoa beans across 168 h. Values are presented as the mean ± SEM of fermentation replicates. Significance between time points was determined by one-way ANOVA and Tukey’s HSD post-hoc test (*p* < 0.05). Time points with different letters are significantly different values.

Concentrations of fermentation substrates and metabolites from the beans are shown in Figure 4. Fructose, glucose and citric acid concentrations rose significantly over the first 48 h before peaking and then quickly decreasing. Succinic, lactic and acetic acid fluctuated before reaching maximum concentrations at 120 h, followed by decreasing levels for the final 48 h of fermentation. Sucrose is the only compound that showed a quick and sharp decrease in concentration. After a 96-h decline, sucrose concentrations ended at undetectable levels. Bean alcohol trends were like those of the simulated pulp media, with ethanol and glycerol remaining constant for 24 h before demonstrating a sharp increase. Glycerol concentrations fluctuated more than those of ethanol, peaking at 96 h compared to ethanol’s 48 h maximum concentration.

### 3.3. Total Polyphenol and Flavanol Content

**Figure 5:**
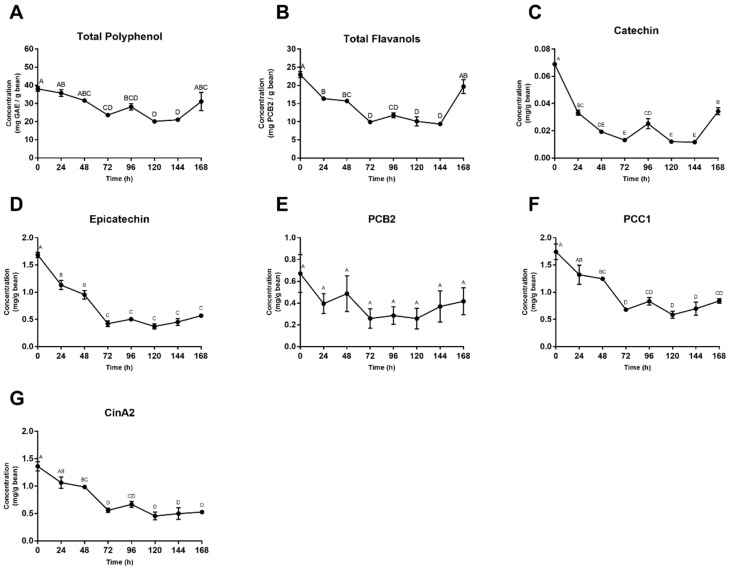
(**A**) Concentration of total polyphenols over the 168-h fermentation, as quantified by the Folin–Ciocalteu colorimetric assay, expressed in mg GAE/g cocoa bean. (**B**) Concentration of total flavanols over the 168-h fermentation, as quantified by the 4-dimethylaminocinnamaldehyde (DMAC) colorimetric assay, expressed in mg PCB2/g bean. (**C**–**G**) Individual polyphenol concentrations (C, EC, PCB2, PCC1, CinA2) over the 168-h fermentation, as quantified by reversed phase UPLC-MS, and expressed as mg/g cocoa bean. Values are presented as the mean ± SEM of fermentation replicates. Significance between time points was determined by one-way ANOVA and Tukey’s HSD post-hoc test (*p* < 0.05). Time points with different letters are significantly different within values.

Mean total polyphenol levels were initially 38.0 mg gallic acid equivalents (GAE)/g bean and after 168 h finalized at 31.1 mg GAE/g bean, for a total loss of 18.3%. Yet after 120 h, there was a more significant net polyphenol loss, quantified at 47.1% loss from initial levels. This difference can be attributed to the 35.3% apparent polyphenol gain in the final 48 h, 120–168 h (Figure 5A). When looking at total flavanol concentrations in Figure 5B, a similar trend can be seen in the last day of fermentation. A 14.4% loss in flavanol concentration is accounted for when looking at initial and final hours (0 and 168 h), whereas a 59.3% loss is seen between 0 and 144 h, with this difference being attributed to the apparent 52.5% gain in total flavanol concentration between the final 24 h (Figure 5B).

### 3.4. Individual Polyphenol Analysis

Concentrations of (±)-catechin, (–)-epicatechin, PCB2, PCC1 and CinA2 are shown in Figure 5C–G. (–)-Epicatechin levels decreased the most, with 66.2% of initial concentrations lost between 0 h and 168 h. CinA2 concentrations fell by 61.3%, followed by PCC1 at 51.8%, catechin at 51.3% and PCB2 at 38.0%. Each individual compound mirrored the trend seen in total polyphenol concentrations in the final 48 h (120–168 h). The most significant loss in all compounds was within the first 48–72 h, followed by fluctuating values until the final 48 h (120–168 h), where all compounds then increased. 

**Figure 6:**
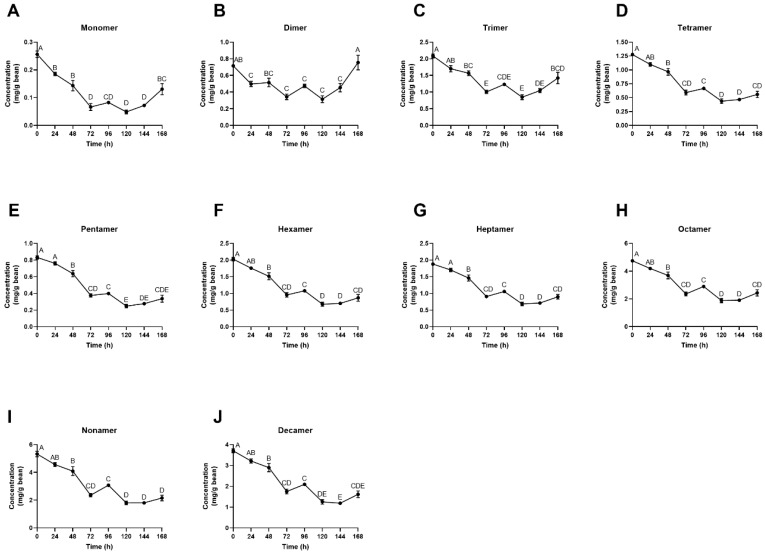
(**A**–**J**) Individual polyphenol concentrations by mean degree of polymerization (mDP) over the 168-h fermentation as quantified by HILIC UPLC-MS/MS and expressed as mg/g cocoa bean. Values are presented as the mean ± SEM of fermentation replicates. Significance between time points was determined by one-way ANOVA and Tukey’s HSD post-hoc test (*p* < 0.05). Time points with different letters are significantly different within values.

Concentrations of individual procyanidins analyzed by degree of polymerization (DP) from monomer through decamer mirrored trends seen previously in that significant losses occurred in the first 72 h, followed by an increase in concentrations of all compounds from 120–168 h. Dimer concentration (Figure 6B) increased over 2-fold from 120–168 h, returning to initial (0 h) concentration levels by the end of fermentation. As expected, monomers (Figure 6A) had the greatest loss with an 80% decline in native compounds from 0–120 h. All other compounds had losses between 42–63% in the first 120 h of fermentation, followed by increases in the final 48 h (120–168 h).

## 4. Discussion

The primary goal of this work was to develop and characterize a pilot-scale model cocoa fermentation system suitable for conducting cocoa fermentation research in the absence of fresh cocoa pods, and capable of producing sufficient quantities (tens of kg) of material for further evaluating the impact of cocoa fermentation on putative bioactive compounds and cocoa bioactivity in in vitro and in vivo experiments. Our model was not designed to physically mimic a scaled-down heap fermentation, but rather to serve as a model system suitable for the study of heap fermentation using (1) dried, unfermented beans and a simulated liquid pulp media as the starting material, (2) ambient microbiota and (3) regular stirring. The goal was to achieve similar chemical changes as observed in on-farm heap fermentations by putting beans in similar conditions to those found at the center of a well-turned heap. In cocoa fermentations conducted on farms, highly variable conditions exist between regions, countries, farms and even between specific heaps, as temperature and environment play important roles in fermentation [44]. Using separate boxes under identical conditions provided some insight into the amount of variability to expect in this controlled model. Variability within our model was generally very minor and the metabolic profiles were similar to those seen in heap fermentations. Although our model was consistent, the replicate fermentations were conducted simultaneously in the same incubator. Further work is needed to determine the consistency between batches conducted at different times and in different incubators.

At an initial pH of 3.6, the pulp media created a favorable environment for yeasts to proliferate within the first 48 h [12]. Citric acid was then metabolized by LAB and ethanol production continued, increasing the pH and encouraging the growth of LAB. As lactic and acetic acids dominated the system, pH declined (72–168 h) and bean cotyledon was penetrated to initiate bean death, where endogenous biochemical reactions began the formation of the characteristic chocolate flavor, and pH concluded at approximately 4.6 [45]. In heap fermentations, turning patterns are a primary factor in pH variability. On average, heaps that are turned at least twice progress from pH 3.9–4.6 [8,12,14,34]. Our system was stirred every 12 h to mimic conditions in the center of a well-mixed heap, and our observed pH values align with previously reported values. DO values stabilized after 24 h as the lag phase of yeast metabolism ended. Likely due to a lack of monitoring equipment, there are no published data regarding DO progression in heap fermentations. Moving forward, aeration in the model could be increased to elevate DO. It would be worthwhile to monitor DO in various on-farm fermentation systems to determine accurate values for modeling a given system. Additionally, comparison of our measured FI values with reported values provide evidence that sufficient fermentation can be achieved under these pilot-scale conditions. Anthocyanins and catechin monomers polymerize during fermentation, rapidly disappearing from the bean cotyledon [2,24]. This can also be observed in the cut test. In traditional cut tests of ≥300 beans, bean color should uniformly progress from purple to brown throughout the fermentation, showing the effect of polyphenol oxidase and other reactions that reduce the appearance of color within the bean. Criollo beans have low anthocyanin levels and as such the cut test and FI for these beans does not follow the pattern typically seen with other cultivars such as Trinitario and Forastero [46,47,48,49,50]. However, it is important to include these tests in reference to typical cocoa fermentation quality checks, and future work is needed using this model with other cocoa cultivars.

Yeasts proliferate during the early stages of fermentation, consuming available sugars and converting them to ethanol and carbon dioxide. Sucrose concentrations exhibited a decline in both pulp media and bean, while pulp glucose and fructose concentrations had a 24-h lag period, and bean concentrations of these sugars increased in the beginning hours, likely due to diffusion from the pulp media into the bean. The early decrease in sucrose is likely due to yeast-derived invertase that hydrolyze sucrose into glucose and fructose during the lag period, before the yeasts begin to consume these sugars. Although sugar concentrations vary between replicates, these trends are similar to those seen in traditional heaps [8,16,51]. Ultimately, yeasts consumed all available substrates for growth, inducing inhibition of their own activity, and LAB began to dominate the system with citric acid degradation, subsequently increasing pH and creating an optimal environment for bacterial growth [52]. AAB then thrived in this newly aerobic, less-acidic environment (48–72 h), facilitating the oxidation of ethanol to acetic acid and, further, to carbon dioxide and water, ultimately resulting in bean death. Although the reactions that occur within the bean itself are not well understood, reported consumption and production of metabolites during industrial cocoa fermentation follow a similar pattern to that of the simulated pulp media in the model system described in this study.

During the first days of fermentation, polyphenols are oxidized via polyphenol oxidase and condense into high molecular weight tannins and other complex compounds. These reactions occur as polyphenols, such as (–)-epicatechin, diffuse out of the bean cotyledon and into the media, subsequently aligning with bean death [9,24]. Criollo beans have been reported to have approximately two-thirds of the total polyphenol content of Forastero and Trinitario varieties, yet other studies have indicated that Criollo beans have high levels of procyanidins with no significant difference in total polyphenol content between the three main cultivars [40,53,54]. Total phenolic content of Criollo beans is often not thoroughly analyzed, but values of 40–50 mg GAE/g have been reported, aligning with the data presented in this study (Figure 5A) [53]. The most dramatic decrease in phenolic concentrations took place in the first 72 h (Figure 5 and Figure 6), confirming this model system’s activity and succession of bean death, as well as representing the quick decline of polyphenol compounds seen in Criollo bean fermentation [40]. Additionally, the observed loss of polyphenols from raw to fermented is comparable to that of heap fermentations. The average loss of total polyphenol content previously reported is 40–60%, which is within the range of loss for this study [3,9,17,23,46,55,56]. Payne et al. [55] found that heap fermentation resulted in an 86–94% decrease in (–)-epicatechin and an 83–89% decrease in (±)-catechin levels. Similarly, Kim and Keeney [23] demonstrated that (–)-epicatechin levels declined 77–91% during fermentation, with bean origin and variety playing a key role in rate and total decline. Furthermore, when examining polyphenols based on degree of polymerization, Kealey et al. [30] reported losses of 61% (monomer), 54% (dimer), 60% (trimer) and 68% (tetramer). Although these values align with our data, that fermentation concluded at 120 h, and it cannot be determined if the trend seen in the final 48 h of our model system would correspond with observations from that study. The quantification of high molecular weight procyanidins (trimer-decamer) over the course of fermentation (Figure 6) is the first of these values to be reported in the literature, as standards are often not commercially available. This data shows the elevated presence of very large molecular weight compounds (Figure 6I–K) throughout fermentation and highlights the research gap on these compounds in cocoa, with most of the literature focusing on monomeric compound quantification, specifically (–)-epicatechin and (±)-catechin.

**Figure 7:**
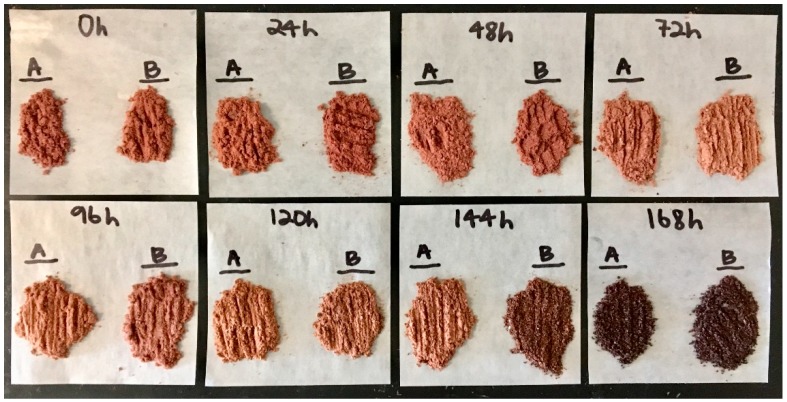
Polyphenol-rich cocoa extracts (CE) prepared at each point throughout the 168-h fermentation. See Section 2.8 for methodology. It is important to note the change in color and texture towards the final hours of fermentation.

Elevated temperatures of the fermentation system may also be responsible for phenolic degradation, with further polymerization of these smaller monomers and procyanidins into larger more complex compounds [55]. These could form complex compounds with higher responses in our assays, but further investigation into the relationship between fermentation temperature and polyphenol loss is warranted as this theory has not been adequately investigated. Figure 7 shows the visual appearance of the polyphenol-rich cocoa extract used for the quantification of total/individual polyphenols and total flavanols. At 144 h, a change in the extract can be noted in the color and texture. Because the four different analyses (Folin, DMAC, UPLC-MS, UPLC-MS/MS) were performed at different times and produced similar results in terms of time-course trends, it is unlikely that the phenolic increase in the final hours is due to human error during analysis. An error in extract preparation is possible but also unlikely, as the appearance of the cocoa extracts change in multiple instances. Further investigation into a wider range of compounds is warranted to determine the cause of this phenolic increase, as it is inconsistent with any previously reported data and there is a lack of thorough data quantifying these individual compounds across varying degrees of fermentation. The mechanism by which these late-stage changes occurred warrants further study. It is important to note that the specific beans used for the curt test (Figure 1C) were not the same actual beans used for extraction and other assays, and furthermore that extraction isolates and concentrates components that cannot always be visibly observed in beans.

Although polyphenol loss may have negative implications on the overall health benefits of cocoa, this relationship is still poorly understood. It is important to understand that these losses correspond with the development of positive flavor profiles, and a balance must be found between optimization of polyphenol content for health outcomes and for acceptable flavor [3]. Furthermore, the chemistry of these losses, and the structures and bioactivity of the subsequent products, remain to be elucidated.

Interest in controlled fermentation systems has emerged as a strategy to experimentally manipulate cocoa fermentation on various scales in regions where cocoa does not grow. Controlled fermentations using a model system offer reliable, reproducible methods to understand the microbial and biochemical reactions that occur. In heap fermentations, beans and pulp are removed directly from the pod, heaped on the ground or in wooden boxes and covered with banana leaves. Traditional systems, while effective for cocoa production, offer little ability to implement experimental conditions or controls to enable research on the impact of environment, materials and fermentation management practices on outcomes of fermentation. In a controlled setting, factors such as temperature, bulk density, relative humidity, oxygenation, microbial inoculation and other influences can be regulated and manipulated, furthering scientific understanding of the complex interactions occurring during cocoa fermentation. Additionally, previously published fermentation-like model systems range in capacity from 25 beans to 1.2 kg rehydrated bean weight [19,22,57]. With the capacity of our model to ferment approximately 30 kg of rehydrated cocoa beans simultaneously, this novel pilot-scale model can produce relatively large quantities of fermented beans in a controlled setting. Production of a larger quantity of material under controlled experimental fermentation conditions will enable further study of the bioactivity of the resulting cocoa, using animal feeding experiments, for example. Furthermore, this pilot-scale model fermentation can be conducted using food-grade inputs, making it applicable to the production of substrates for human clinical trials as well, where large amounts of material are required.

It is critical to note that the system developed in this study is intended to serve as a model designed to replicate the chemical outcomes of cocoa fermentation, but not to physically duplicate the heap fermentation process on a smaller scale. As such, our model aims to produce conditions mimicking the center of a well-turned heap in simulated pulp media. Our data indicate that this system is a reliable and controlled pilot-scale fermentation model, which replicates the outcomes of heap fermentation with acceptable fidelity. It is important to note that cocoa fermentation varies significantly. Our model system was not tested in the present study to mimic all possible combinations of turning, aeration, temperature gradients or lengths of fermentation. However, the model we describe here can be used to study such variations in a controlled environment. We are currently expanding upon this study by introducing variables such as cool vs. hot fermentations to generate cocoas with distinct chemical profiles for evaluating the impact of composition on health benefits. Although our results show promise moving forward, this model is not without limitations. The beans used were commercially available and of largely unknown origin. Although the beans were food-grade and declared to be dried and unfermented, drying temperature and duration, as well as the conditions between harvest and drying, are unknown. Moving forward, we will address this limitation by obtaining dried unfermented beans from suppliers with more knowledge of the supply chain. The impact of using dried, unfermented beans on the fidelity of our model, compared with fermentation using fresh beans, will need to be evaluated by using a single batch of beans and conducting fermentations using fresh beans, as well as by drying and then employing our model. For example, early flavanol degradation by endogenous polyphenol oxidase may be impacted. Also, aeration could be further optimized to mimic conditions at the center of a well-mixed heap or box fermentation, or at least to gain an understanding of the effects of aeration on the chemical changes observed. Acquiring fresh cocoa pods for use in laboratories distant from production regions without spoilage has proven to be expensive and ineffective.

To summarize the novelty of the present work, several key advances have been made. First, by using dried, unfermented beans and a simulated pulp medium instead of relying on fresh cocoa pods, this model system will allow cocoa fermentation research to progress anywhere in the world regardless of location, climate or season. This greatly expands the ability of researchers distant from cocoa cultivation to control and manipulate cocoa fermentation for research purposes. Second, the increased scale of this model system (from previous controlled lab fermentations using tens or hundreds of grams of beans to the present model employing 2 × 15 kg beans) will facilitate production of large amounts of custom cocoas designed specifically for animal or human clinical studies. Both of these advances will result in expanded scope of cocoa fermentation research. Thus, the development and characterization of this pilot-scale model system represents a promising new and controlled method for expanding upon cocoa fermentation research.

## Figures and Tables

**Table 1:** MS settings for individual polyphenol analysis by reverse-phase (RP)-UPLC-MS.

Compound	*t*_R_ *^a^*	[M–H]^–^ *^b^*
	(min)	(m/z)
(±)-catechin	2.946	288.95
(–)-epicatechin	3.625	289.01
PCB2	3.366	576.84
PCC1	3.904	864.85
CinA2	4.063	1153.19

*^a^* Retention time *^b^* QDA detector uses singly charged parent ions for selected ion response (SIR) monitoring.

**Table 2:** Tandem MS/MS settings for multi-reaction monitoring (MRM) detection of monomeric-decameric flavanols.

Compound	*t*_R_ *^a^*	MW	[M–H]^– *b*^	Daughter Ion
	(min)	(g mol^−1^)	(m/z)	(m/z)
Monomer	0.61	290.27	289.03	245.06
Epigallocatechin	0.74	458.37	305.04	124.98
Dimer	2.03	578.52	577.14	425.10
Trimer	3.05	866.77	865.22	287.07
Tetramer	3.73	1155.02	576.40	125.02
Pentamer	4.26	1443.28	720.41	125.02
Hexamer	4.66	1731.53	864.52	125.02
Heptamer	5.00	2017.81	1008.40	125.17
Octamer	5.28	2308.03	1152.58	125.17
Nonamer	5.53	2596.54	864.12	125.17
Decamer	5.75	2884.54	960.18	125.17

*^a^* Retention time. *^b^* All MRMs used singly charged parent ions except for pentamer, hexamer, heptamer and octamer, which are double-charged ([M–2H]^2−^), and nonamer and decamer, which are triple-charged ([M–3H]^3−^).
